# Da-Chuan-Xiong Decoction Ameliorates Sodium Sensitivity and Plasma Norepinephrine via Attenuation of Brain Oxidative Stress in the DOCA-Salt Hypertensive Rats

**DOI:** 10.1155/2024/2226143

**Published:** 2024-02-02

**Authors:** Qi Zhang, Hao Li, Shan Zhao, Fangfang Li, Yingying Tan

**Affiliations:** ^1^Shaanxi Key Laboratory of Chinese Medicine Encephalopathy, Shaanxi University of Chinese Medicine, Xianyang 712046, China; ^2^School of Basic Medical Science, Shaanxi University of Chinese Medicine, Xianyang 712046, China

## Abstract

**Background:**

Da-Chuan-Xiong Decoction (DCXD) is an aqueous extract from a classic Chinese herbal formula composed of dried rhizomes of Ligusticum chuanxiong Hort and *Gastrodia elata* Bl. in the mass ratio of 4 : 1. It has been long used to treat chronic cardiovascular disease caused by blood stasis and wind pathogen in the clinic. This experimental study aimed to investigate the blood pressure (BP)-lowering effect of DCXD treatment on hypertension and underlying mechanisms.

**Methods:**

Male Sprague–Dawley rats were used in the experiment, and the hypertensive models were created by administering deoxycorticosterone acetate (DOCA) in conjunction with a high salt intake in uninephrectomized rats. DCXD was administered to hypertensive rats by oral gavage daily at a dose of 5 g/kg or 2.5 g/kg bodyweight for 28 days. The brain sodium sensitivity, ENaC function, superoxide anion level, NADPH oxidase activity, and expression of ENaC, p67phox, p47phox, and Rac1 in the paraventricular nucleus were assessed by using the appropriate methods.

**Results:**

The 28 days of DCXD (5 g/kg) treatment significantly reduced the increased BP effectively, inhibited the enhanced heart index, kidney index, and 24 h urinary protein, and improved the progressive pathological changes of heart and kidney, which was comparable to that of the positive control amlodipine. DCXD treatment also caused a marked reduction in plasma norepinephrine and induced a significant improvement in brain sodium sensitivity and ENaC function in DOCA-salt hypertensive rats. Rats in DCXD-treated groups also exhibited decreased superoxide anion levels and NADPH oxidase activity in the paraventricular nucleus. The level of ENaC, p67phox, and Rac1 protein expression in the paraventricular nucleus was significantly downregulated by DCXD treatment in DOCA-salt hypertensive rats.

**Conclusions:**

These findings indicate that the depressor action and sympathetic inhibition of DCXD on salt-sensitive hypertension may be by ameliorating brain sodium sensitivity, modulating ENaC function, and inhibiting the expression of ENaC and NADPH oxidase in the hypothalamic paraventricular nucleus.

## 1. Introduction

Worldwide, hypertension is the leading cause of cardiovascular disease and premature death. Although increasing antihypertensive drugs are available, rates of awareness, treatment, and control of hypertension are unacceptably low [1]. The main reason is that hypertension is a multifaceted disease caused by a complex interplay of environmental and genetic factors. High salt intake is known as the most important environmental factor, and salt sensitivity is the common phenotype of hypertension [2]. Studies have shown that high salt intake activates central autonomic circuitry leading to exaggerated sympathetic activity, which is a major contributor to the pathogenesis of salt-sensitive hypertension, and modulation of sympathetic overactivity is an important target for hypertension treatment [3]. Among the central neural networks controlling sympathetic activity, the hypothalamic paraventricular nucleus (PVN) is identified as the pivotal integration area [3]. It not only receives cardiovascular information from arterial baroreceptors, atrial volume receptors, and central osmoreceptors but also contains neurons that release vasopressin and innervate the sympathetic preganglionic neurons [4]. Accumulating evidence from experimental models and humans with hypertension and heart failure have demonstrated that augmented neuronal activation of the PVN contributes to increased sympathetic nerve activities at the level of the end-organ and sodium and fluid retention [4]. Thus, improving the abnormal neuronal activity of PVN is probably an important way to reduce sympathetic activity and prevent salt-sensitive hypertension.

Traditional Chinese herbal medicine has been widely applied in various disorders, including cardiovascular disease in Asia for over 2,000 years as a complementary treatment. In Chinese herbal therapy, the most widely used herbal formulas are combined with many herbs and prepared according to traditional Chinese formulation concepts. Da-Chuan-Xiong Decoction (DCXD), which is composed of two herbs, dried rhizomes of Ligusticum chuanxiong Hort (*Chuanxiong*)and *Gastrodia elata* Bl. (*Tianma*) in the mass ratio of 4 : 1, is a classic Chinese herbal formula that originated from “Xuan Ming Lun Fang” (an ancient Chinese medical book written by Liu Wansu in the Jin Dynasty). It has been applied for treating various cardiovascular and cerebrovascular diseases including hypertension, coronary heart disease, stroke, and migraine caused by blood stasis and wind pathogens for hundreds of years [5, 6]. Several clinical studies indicate that DCXD as the basic prescription in the treatment of hypertension has a clear effect on stabilizing blood pressure, improving the syndrome performance of patients, and ameliorating early target organ damage [6–8]. In the angiotensin II-induced hypertensive model, DCXD was found to reduce arterial blood pressure and downregulate the abnormal expression and functional activity of AT1R-NADPH oxidase in the hypothalamus [9]. In the traumatic brain injury rat model, DCXD was demonstrated to decrease BBB leakage and brain edema and reduce neuron loss, microglia, and astrocyte activation [10]. DCXD has also been reported to exhibit an anti-inflammatory effect in LPS-induced RAW 264.7 cells through the inhibition of the NF-*κ*B pathway. Furthermore, DCXD exhibited neuroprotective effects against focal cerebral ischemia and reperfusion in rats [11]. All these pieces of evidence indicate that DCXD has the potential for neuroprotection, antioxidation, vascular protection, and autonomic nerve regulation. However, the actual mechanism underlying DCXD's beneficial effect is unknown.

The epithelial sodium channel (ENaC) is a cationic channel activated by extracellular protons and widely distributed in renal tissue, blood vessels, and central nervous system that plays a critical role in the pathological development of salt-sensitive hypertension. In the brain, ENaC is demonstrated in neurons of PVN, supraoptic nucleus, and subfornical organ, and choroid plexus [12]. In several animal models of hypertension and heart failure, functional studies have shown that central administration of the ENaC blocker eliminates sympathetic hyperactivity and hypertension [12]. The expression and activity of ENaC within PVN neurons are also increased in Dahl salt-sensitive rats during high dietary salt intake [13]. These studies suggested that ENaC in the brain plays a key role in both neuroendocrine and autonomic responses, and modulation of ENaC activity in the neurons may be an important way to prevent the development of salt-sensitive hypertension [13]. However, whether alterations in ENaC activity are involved in the protective effect of DCXD is unknown.

NADPH oxidase is a multisubunit enzyme that catalyzes the reduction of molecular oxygen to form reactive oxygen species. It includes membrane subunits p22phox and gp91phox as well as cytoplasmic subunits p47phox, p67phox, and p40phox and the small G protein Rac1 [14]. Numerous studies have shown that genetic and pharmacological inhibitions of NADPH are neuroprotective and are able to reduce detrimental aspects of pathology following hypertension and heart failure. Immunohistochemical studies indicated that NADPH subunits are widely distributed in the hypothalamus, and these sites also express modulation proteins of ENaC [15]. Our recent studies found that DCXD had a significant inhibitory effect on abnormal sympathetic activities and brain NADPH oxidase expression in hypertensive rats induced by angiotensin II [9]. It has also been demonstrated that NADPH oxidase-mediated reactive oxygen species formation is an important way to activate ENaC function [16]. Therefore, the activity and subunit expression of NADPH oxidase in the hypothalamus were evaluated to investigate the potential role of NADPH oxidase in the inhibitory effects of DCXD treatment on brain ENaC activity. DOCA-salt hypertensive rat is a common salt-sensitive model of hypertension characterized by neurohumoral activation which is induced by chronic DOCA administration, reduced renal mass, and a high salt diet [17]. The purpose of this study was to assess whether the BP-lowering and sympathoinhibitory effect of DCXD treatment on DOCA-salt sensitive hypertension was related to modifications in ENaC function and NADPH oxidase within the PVN of hypertensive rats.

## 2. Methods and Materials

### 2.1. Animals

Sixty male Sprague–Dawley (SD) rats, 8 weeks old, weighing 200 ± 10 g, were supplied by Xi'an Jiaotong University's Experimental Animal Center. All rats were raised under well-maintained settings, including a temperature of 22 ± 2°C, humidity of (50 ± 5) %, and 12 hours of alternate light and darkness. Rats were fed and treated in accordance with the US National Academies Press's Guidelines of Care and Use of Laboratory Animals standards (eighth edition, update 2011). The Institutional Animal Care and Use Committee at Shaanxi University of Chinese Medicine approved the protocols (#20200517-2).

### 2.2. Materials and Reagents

Deoxycorticosterone acetate pellets (DOCA, #SM-121) are provided by Innovative Research of America. Isoflurane is the product of Shenzhen Reward Company. Amlodipine besylate tablets (AML, #H10950224) are produced by Pfizer Pharmaceutical Company. Dried rhizomes of Ligusticum chuanxiong Hort (#801003917, chuanxiong) and *Gastrodia elata* Bl (#801002511, Tianma) were provided by Beijing TongRenTang (Bozhou) Co. Ltd.., which were identified by Professor Hu Benxiang of School of Pharmacy, Shaanxi University of Chinese Medicine, as specified varieties in Pharmacopoeia. p67phox antibody (#sc-374510), p47phox antibody (#sc-17845), Rac1 antibody (#sc-514583), ENaC antibody (#sc-48428), and GAPDH antibody (#sc-47724) were provided by Santa Cruz Biotechnology Company. Lucigenin (#sc-202698A) is produced by Santa Cruz Biotechnology Company. NE enzyme-linked immunosorbent assay kit (ELISA, #BAE-5200) is provided by Rocky Mountain Diagnostics Company. Benzamil (#B2417) and hexamethonium (#H2138) are provided by Sigma-Aldrich.

### 2.3. DCXD Compound Preparation and Component Analysis

DCXD is composed of chuanxiong rhizome and *Gastrodia elata*, and the decoction was prepared by the aqueous extraction method. Dried rhizomes of Ligusticum chuanxiong Hort and *Gastrodia elata* Bl were accurately weighed in a 4 : 1 ratio and soaked for 2 h in 10 times the volume of distilled water. The soaking mixture was boiled twice for 1 h and filtered. The twice decocted filtrates were combined and concentrated at 60°C to contain 1 g/mL of raw herbs. The prepared decoction was stored at 4°C and diluted to two different concentrations (5.0 and 2.5 g of crude herb/kg body of weight) in water before being used in animal experiments.

To analyze the composition of prepared DCXD, ultra-performance liquid chromatography to quadrupole time-of-flight mass spectrometry (UPLC-Q/TOF-MS) analysis was performed with ferulic acid and gastrodin as the main active constituents for quality control. The analyzed DCXD samples were diluted with methanol and filtered through a 0.22 *µ*m microporous membrane. Waters H-Class UHPLC (Waters) coupled with AB Sciex TripleTOF® 4600 mass (AB SCIEX) was used to analyze the DCXD extracts. The extract was separated on an Agilent ZORBAX RRHD Eclipse XDB-C18 (2.1 × 100 mm, 1.8 *µ*m) at 30°C, and the mobile phase was acetonitrile (*A*) and 0.1% formic acid aqueous solution (*B*). The mass spectrometer analysis was performed with electrospray ionization (ESI) negative and positive ion modes. The mass spectrometry data were collected and analyzed by Analyst TF 1.7.1 and PeakView 1.2 software. Compositional identification of each compound was compared to Natural Products HR-MS/MS Spectral Library 1.0 according to the chromatographic peak score information.

### 2.4. Animal Model and Experimental Protocol

**Figure 1:**
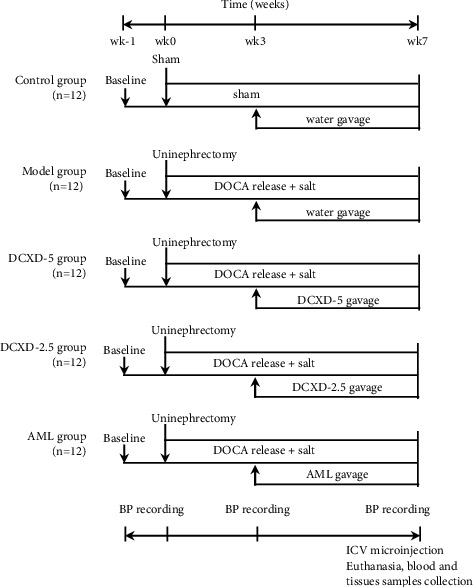
The schematic representation of animal grouping and experimental protocol.

The schematic of the experimental design is shown in Figure 1. The hypertensive model was induced according to a previous study [17]. In brief, rats were allowed to acclimate for 1 week, after which they were anesthetized by pentobarbital sodium (40 mg/kg, ip). Under sterile conditions, a retroperitoneal flank incision was made and the left kidney was removed. DOCA pellets (150 mg/kg) were implanted subcutaneously on the back sides of rats after an incision had been sutured. Model rats received drinking water containing 1% NaCl and 0.2% KCl the day after the surgical procedure. Sham-operated rats received the same surgical procedures without kidney removal as well as DOCA implantation, and they were given normal tap drinking water. After 3 weeks, the hypertensive model was successfully identified if systolic blood pressure (SBP) was ≥160 mmHg by tail-cuff technique in comparison to the preoperation [18]. In addition to the sham operation control group (c, *n* = 12), model rats meeting the standard of hypertension were randomly divided into four groups, DOCA-salt model group (model), DCXD-5 high-dose group (DCXD-5), DCXD-2.5 low-dose group (DCXD-2.5), and amlodipine (AML) group, with 12 rats in each group. Then, the rats in the DCXD-5 group and the DCXD-2.5 group were orally administered with DCXD (5 g/kg/day and 2.5 g/kg/day), and rats in the AML group were given amlodipine (1 mg/kg/day, dissolved in pure water) by gavage for 4 weeks. Throughout the experiment, both the model and control groups received the same volume of pure water by gavage.

This animal experiment lasted for 8 weeks. Blood pressure was monitored weekly using a tail-cuff technique. In each group of experimental animals (*n* = 12), six rats were randomly selected for intracerebroventricular (ICV) microinjection, and the remaining rats were used to collect urine, blood, and tissue specimens. At the endpoint of the experiment, urine collection was performed using metabolic cages over 24 h to measure proteinuria and urine creatinine. The rats were then anesthetized, and abdominal blood was drawn for plasma creatinine and norepinephrine (NE) measurements. Under anesthesia with an overdose of isoflurane, the rats were killed. On the ice plate, brain tissue was quickly dissected out and the bilateral PVN tissues were obtained by punch-out technique, which were frozen quickly in liquid nitrogen for Western blot analysis. Meanwhile, kidney and heart tissues were harvested, weighed, and placed in 4% paraformaldehyde for hematoxylin and eosin staining.

### 2.5. BP Determination

For tail-cuff measurement, the CODA noninvasive BP acquisition system (Kent Scientific Corporation, Torrington, CT, USA) was used to measure conscious artery BP. Before baseline BP measurements, rats were kept in a specified quiet location and better adapted to restraint and tail-cuff inflation for 5 days. The occlusion cuff was positioned at the base of the tail and inflated to 250 mm·Hg before being deflated over a 20-second period. The software system tracked and analyzed the systolic blood pressure (SBP), diastolic blood pressure (DBP), heart rate (HR), and mean arterial pressure (MAP). The blood pressure was assessed once a week between 10 : 00 AM and 11 : 00 AM for each rat and was repeated three times. The mean value was calculated as the BP of the rat this week. The experiment was carried out by the same person over the same time period.

### 2.6. Biochemical Detection

Rats in each experimental group (*n* = 6) were placed in metabolic cages 48 h before the end of the experiment, collecting the last 24 h of urine. After collecting urine, blood was drawn from the abdominal aorta after anesthesia. After centrifugation, an automatic biochemical analyzer was used to detect plasma creatinine, 24 h urinary protein, and creatinine clearance (ml/min) = urine creatinine (mmol/L) × 24 h urine volume (mL/min)/plasma creatinine (mmol/L) × 1440. Plasma NE levels were quantified using a commercially available rat ELISA kit according to the manufacturer's instructions.

### 2.7. Intracerebroventricular (ICV) Microinjection

The animals (*n* = 6 in each group) were anesthetized with 2% isoflurane inhalation at the end of the four-week treatment. During ICV microinjection, invasive BP recording was carried out to monitor beat-to-beat BP fluctuations by direct methods, which is the gold standard for accurate BP measurement. For direct arterial BP recording and intravenous operation, the left femoral artery and vein were cannulated with polyethylene catheters filled with heparin solution. Following the aforementioned procedure, the arterial cannula was attached to a pressure transducer connected to an amplifier (FE221, ADInstruments, Bella Vista, NSW, Australia). The ADInstruments PowerLab data acquisition system (Bella Vista, NSW, Australia) was applied to collect and analyze arterial blood pressure signals.

The rat was placed in a stereotaxic apparatus while being inhaled with 3% isoflurane. Following surgical brain exposure, a glass microinjection pipette (tip size: 100 *μ*m) linked to a microinjecting system was positioned and placed in the right lateral ventricle. The right lateral ventricle is 1.0 mm caudal from the bregma, 1.4 mm lateral to the midline, and 4.5 mm ventral to the dura, as determined by the Paxinos and Watson rat atlas. Following the baseline assessment of MAP and HR, either high-Na (0.2 mol/L) or regular-Na (0.145 mol/L) aCSF was infused ICV with a microsyringe pump at a flow rate of 1 L/min for 10 minutes. Furthermore, benzamil is a known inhibitor of the ENaC. To evaluate brain ENaC function among the groups at the end of the experiment, the cardiovascular responses to ICV injection of benzamil (1 mmol/L and 1 *μ*L/min for 5 minutes) were also assessed in the protocol. The dose of each chemical was defined according to the previous reports [19]. During experimental observation, the body temperature of the rat was maintained from 36.5°C to 37.5°C by a heating pad. 2% Evans blue (100 nL) was used for histological identification of the microinjection site. The experimental data of microinjection sites out of the ICV were excluded from the analysis.

### 2.8. Assessment of Plasma Norepinephrine

The plasma NE levels were assessed by employing the ELISA method and evaluating blood pressure responses to the ganglionic blockade. At the end of the experiment, the ganglionic blocker hexamethonium (30 mg/kg, iv) was chosen to check the resting sympathetic vasomotor tone, and the recording changes of MAP were utilized as an index of sympathetic activity [18]. During the experiment (*n* = 6 in each group), BP was recorded for 20 min both before and after hexamethonium injection, and the maximum fall in BP was analyzed.

### 2.9. Detection of Hypothalamic Superoxide Anion and NADPH Oxidase Activity

Under anesthesia with an overdose of isoflurane, the rats (*n* = 6 in each group) were killed at the end of the experiment. On the ice plate, brain tissues were quickly dissected out and sliced into 500 *μ*m coronal sections. The punch-out technique was employed to obtain the bilateral PVN tissues. The supernatant was obtained after homogenizing and centrifuging the tissues for subsequent analysis of superoxide anion and NADPH oxidase activity. Protein concentration was determined using the Bradford method. The superoxide anion level was determined by using lucigenin as a chemiluminescence reagent. The supernatant of the sample to be tested was diluted with the modified HEPES buffer. The sample was added with lucigenin (5 *μ*mol/L) and incubated at 37°C for 10 min. The luminescence value was determined by a SpectraMax M5 multifunctional microplate reader, and the mean light unit (MLU) per milligram of protein per minute was calculated. NADPH oxidase activity was checked by the chemiluminescence method to determine the level of superoxide anion. The supernatant of the sample was diluted in the modified HEPES buffer, and NADPH (100 *μ*mol/L) and lucigenin (5 *μ*mol/L) were added. The luminescence value was detected by a multifunctional enzyme labeling instrument, and the average luminescence value of each milligram protein per minute was calculated. The background chemiluminescence in buffer containing lucigenin (5 *μ*mol/L) was also determined according to previous studies [9].

### 2.10. Histological Analysis

The heart and kidney tissues were embedded in paraffin after being fixed with 4% paraformaldehyde and were then cut into 10 *µ*m thick sections, and the transverse sections were stained with hematoxylin and eosin. The histological changes of tissues in each group were examined under a microscope and assessed using Image J analysis software by two to three blinded researchers.

### 2.11. Western Blot

Protein expression in PVN homogenates was analyzed by Western blot analysis. PVN tissues (*n* = 6 in each group) were collected as described above and homogenized in an ice-cold lysing buffer. The tissue lysate was centrifuged, and the supernatant was collected. The protein concentration was determined by using the Bradford protein quantification kit. Then, the sample with 25 *μ*g of total protein was prepared and loaded in the well of SDS-PAGE gel, and the proteins were separated by electrophoresis and subsequently transferred onto nitrocellulose membranes. After blocking for 1 hour at room temperature with blocking buffer, membranes were incubated overnight at 4°C with the primary antibody, p67phox (1 : 500), p47phox (1 : 500), Rac1 (1 : 500), ENaC (1 : 500), GAPDH (1 : 500), or *β*-actin (1 : 1000). The membranes were then incubated for 1 hour at room temperature with the secondary antibody, horseradish peroxidase-conjugated IgG. Enhanced chemiluminescence autoradiography was used to detect the immunoreactivity on membranes, and the blot images were collected and analyzed using Quantity One software (Bio-Rad).

### 2.12. Statistical Methods

SigmaPlot 12.5 software was utilized to analyze all data, which were expressed as mean ± SEM. For group comparisons, one-way ANOVA and the Newman–Keuls test were used, with *P* < 0.05 considered as statistically significant.

## 3. Results

### 3.1. Components Analysis of DCXD

UPLC-Q/TOF-MS was used to characterize the chemical components of DCXD. The total ion chromatograms (positive and negative) of DCXD are shown in Supplementary Figures 1A–1C. According to the multistage mass spectrometry information of the samples, combined with the high-resolution mass spectrometry database of natural products, a total of 32 compounds were identified from the DCXD samples (Supplementary Table S1).

### 3.2. Effects of DCXD Treatment on Arterial BP, HR, and Pathological Changes of Heart and Kidney in DOCA-Salt Hypertensive Rats

**Figure 2:**
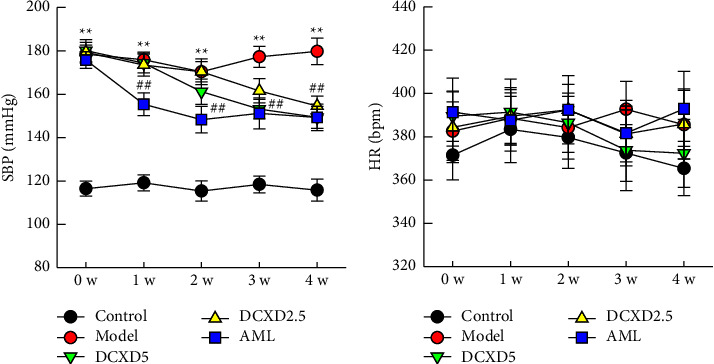
Effects of DCXD on systolic blood pressure (SBP) and heart rate (HR) in DOCA-salt hypertensive rats: (a) change of SBP in different experimental groups and (b) change of HR in different experimental groups. BP and HR were measured in conscious rats by the tail-cuff method. Data are presented as mean ± SEM (*n* = 12). ^*∗∗*^*P* < 0.01 vs. the control group and ^##^*P* < 0.01 vs. the model group.

Tail-cuff measurements were used to measure conscious artery BP before and during the DCXD treatment. As shown in Figure 2, the model group had significantly higher systolic blood pressure (SBP) than the control group (*P* < 0.01). After 4 weeks of DCXD treatment, rats in the DCXD-5 and DCXD-2.5 groups had significantly lower MAP from weeks 3 to 4 than the DOCA-salt group (*P* < 0.05). SBP changes significantly in the AML group 1–4 weeks after treatment. There was no statistically significant difference in HR between the experimental groups (*P* > 0.05).

Heart index, kidney index, and renal function were also examined in all groups of rats. As shown in Table 1, the heart index, kidney index, and 24 h urinary protein in the model group were significantly higher than that in the control group (*P* < 0.01), while the creatinine clearance rate was significantly lower (*P* < 0.01). Compared to the model group, the heart index, kidney index, creatinine clearance rate, and 24 h urinary protein level of rats in the DCXD-5 group, DCXD-2.5 group, and AML group improved to varying degrees (*P* < 0.05), while the effect of DCXD-5 group was more obvious.

**Figure 3:**
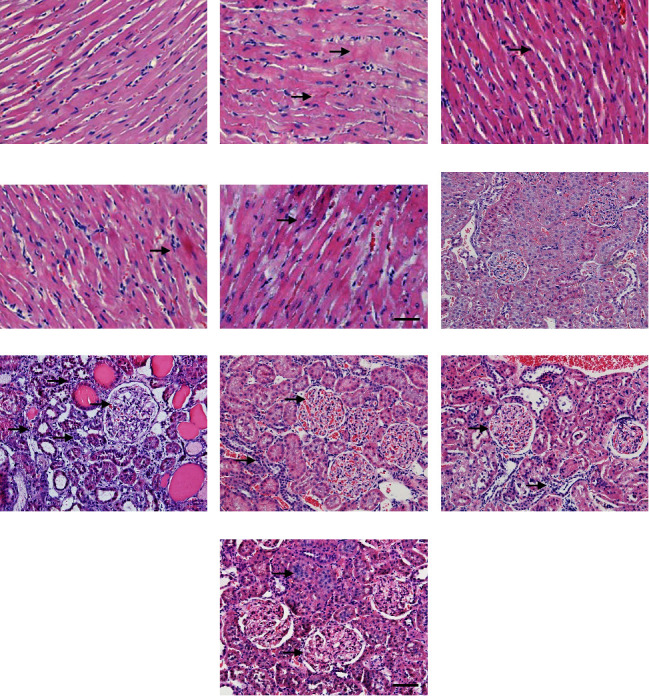
Effects of DCXD on the pathological changes of cardiac and renal tissues in DOCA-salt hypertensive rats. (a–e) HE staining of left ventricular tissues in rats (scale bar = 100 *μ*m), and the arrows point to the myocardial cell hypertrophy, the disordered arrangement of cardiac muscle fibers, and the scattered distribution of the nucleus: (a) control rat, (b) model rat, (c) DCXD-5 rat, (d) DCXD-2.5 rat, and (e) AML rat. (f–j) HE staining of rat renal tissues (scale bar = 100 *μ*m), and the arrows point to the glomerular hypertrophy, basement membrane hyperplasia, and inflammatory cell infiltration: (f) control rat, (g) model rat, (h) DCXD-5 rat, (i) DCXD-2.5 rat, and (j) AML rat.

Figures 3(a)–3(e) show the histopathological changes of left ventricular tissues in rats of each group. In the model group, the myocardial cells were hypertrophic, the arrangement of cardiac muscle fibers was disordered, and the distribution of the nucleus was scattered. In the DCXD-5 group, DCXD-2.5 group, and AML group, the diameter of myocardial cells and the arrangement of cardiac muscle fibers were improved to varying degrees. Figures 3(f)–3(j) show the renal histopathological changes of rats in each group. In the model group, the glomerular hypertrophy, basement membrane hyperplasia, and inflammatory cell infiltration were observed, exhibiting the dilation, atrophy, and vacuolar degeneration of renal tubules and protein tube type. In the DCXD-5 group, DCXD-2.5 group, and AML group, the morphological changes of glomerular and renal tubules and inflammatory cell infiltration were improved to varying degrees.

### 3.3. Effects of DCXD Treatment on Plasma NE in DOCA-Salt Hypertensive Rats

**Figure 4:**
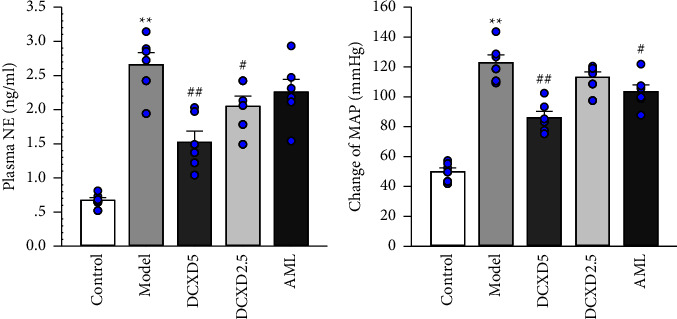
Effects of DCXD on plasma norepinephrine in different experimental groups: (a) plasma NE levels and (b) change of mean arterial pressure (MAP) by injection of ganglionic blocker hexamethonium (30 mg/kg). Data are presented as mean ± SEM (*n* = 6). ^*∗∗*^*P* < 0.01 vs. the control group and ^#^*P* < 0.05 and ^##^*P* < 0.01 vs. the model group.

As shown in Figure 4(a), the plasma NE level increased significantly in the model group when compared to the control group (*P* < 0.01). After four weeks of DCXD treatment, plasma NE levels in the DCXD-5 and DCXD-2.5 groups were significantly lower than in the model group (*P* < 0.01 and *P* < 0.05).

The ganglionic blocker hexamethonium was used to measure the resting sympathetic vasomotor tone. The peak changes of MAP to hexamethonium in the model group were significantly greater than in the control group, as shown in Figure 4(b). The MAP responses of hexamethonium in the DCXD-5 and AML groups improved significantly when compared to the model group (*P* < 0.01, *P* < 0.05), whereas there were no obvious changes in the DCXD-2.5 group.

### 3.4. Effects of DCXD Treatment on Brain Sodium Sensitivity and ENaC Function in DOCA-Salt Hypertensive Rats

**Figure 5:**
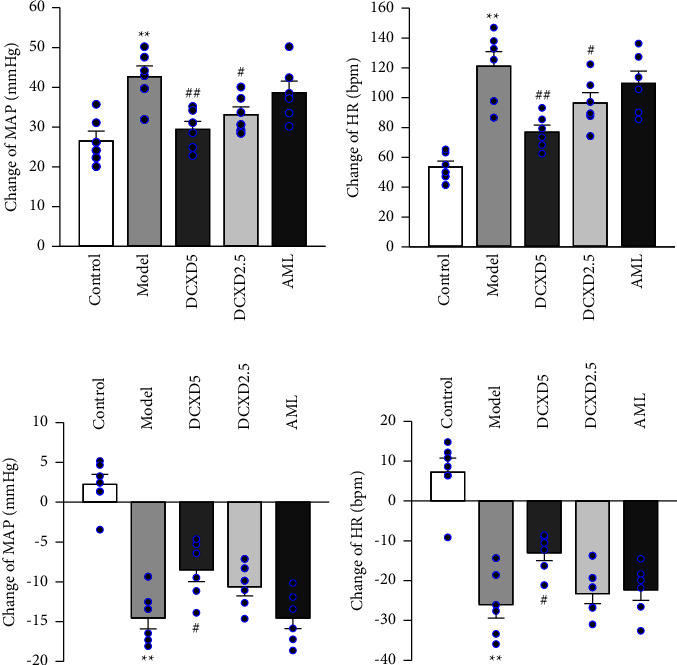
Effects of DCXD on mean arterial pressure and heart rate response to ICV high-Na aCSF and benzamil infusion in different experimental groups: (a) change of MAP to ICV high-Na aCSF infusion (0.2 mol in 10 *µ*L), (b) change of HR to ICV high-Na aCSF infusion, (c) change of MAP to ICV benzamil infusion (10 nmol in 10 *µ*L), and (d) change of HR to ICV benzamil infusion. Data are presented as mean ± SEM (*n* = 6). ^*∗∗*^*P* < 0.01 vs. the control group and ^#^*P* < 0.05 and ^##^*P* < 0.01 vs. the model group.

The baseline MAP and HR were assessed before ICV microinjection in the control group (MAP: 115.82 ± 5.17 mmHg and HR: 365.48 ± 12.53 bpm), model group (MAP: 179.85 ± 6.12 mmHg and HR: 385.67 ± 15.82 bpm), DCXD-5 group (MAP: 149.43 ± 6.15 mmHg and HR: 372.42 ± 15.76 bpm), DCXD-2.5 group (MAP: 154.61 ± 4.58 mmHg and HR: 385.92 ± 16.27 bpm), and AML group (MAP: 149.33 ± 5.12 mmHg and HR: 392.97 ± 17.36 bpm). The arterial BP and HR responses to ICV high-Na aCSF were used to assess the brain sodium sensitivity of each group of rats. High-Na aCSF ICV injection increased arterial BP and HR in both control and model rats, as shown in Figures 5(a) and 5(b), but the degree of these changes was significantly greater in model rats. In comparison to the model group, the MAP and HR changes in the DCXD-5 and DCXD-2.5 groups were significantly lower (*P* < 0.01, *P* < 0.05). Furthermore, the cardiovascular responses to ICV injection of the ENaC blocker benzamil were assessed in order to evaluate the brain ENaC function. Figures 5(c) and 5(d) show that the ICV injection of benzamil significantly decreased MAP and HR in model rats compared to control rats (*P* < 0.01). The changes in MAP and HR in the DCXD-5 group were significantly better than in the model group (*P* < 0.05), whereas no significant changes were observed in the DCXD-2.5 or AML groups.

### 3.5. Effects of DCXD Treatment on the Superoxide Anion Level and NADPH Oxidase Activity in Hypothalamus of DOCA-Salt Hypertensive Rats

**Figure 6:**
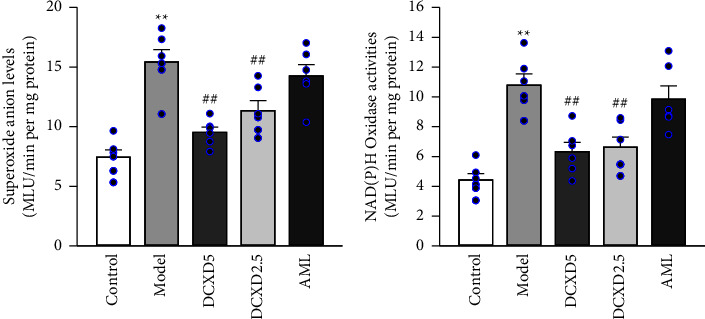
Effects of DCXD on superoxide anions levels (a) and NADPH oxidase activity (b) in the hypothalamus of rats in different experimental groups. The values were expressed as MLU per minute per milligram of protein. Data are presented as mean ± SEM (*n* = 6). ^*∗∗*^*P* < 0.01 vs. the control group and ^##^*P* < 0.01 vs. the model group.

As shown in Figure 6, in the model group, compared to the control group, the hypothalamic superoxide anion level and NADPH oxidase activity were significantly higher (*P* < 0.01). The levels of superoxide anion and NADPH oxidase activity in the hypothalamus of rats in the DCXD-5 and DCXD-2.5 groups were significantly lower (*P* < 0.01) than the model group, but not in the AML group.

### 3.6. Effects of DCXD Treatment on NADPH Oxidase and ENaC Expression in Hypothalamus of DOCA-Salt Hypertensive Rats

**Figure 7:**
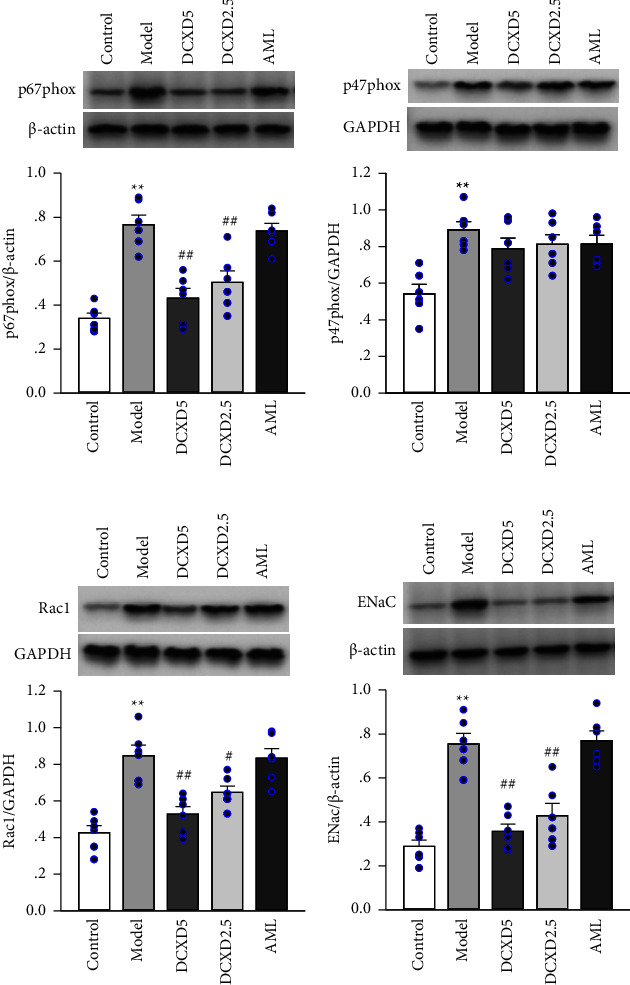
Effects of DCXD on the protein levels of p67phox, p47phox, Rac1, and ENaC in the hypothalamus of each group: (a) protein levels of p67phox, (b) protein levels of p47phox, (c) protein levels of Rac1, and (d) protein levels of ENaC. Relative optical density was measured using Quantity One software and normalized with *β*-actin or GAPDH as a loading control. Data are presented as mean ± SEM (*n* = 6). ^*∗∗*^*P* < 0.01 vs. the control group and ^#^*P* < 0.05 and ^##^*P* < 0.01 vs. the model group.

As shown in Figure 7, Western blot was used to evaluate changes in the expression of NADPH oxidase subunits (p67phox, p47phox, and Rac1) and ENaC in the hypothalamus. The expression levels of p67phox, p47phox, Rac1, and ENaC in the hypothalamus of the model group were significantly higher than that in the control group (*P*  <  0.01). The expression levels of p67phox, Rac1, and ENaC in the DCXD-5 and DCXD-2.5 groups were lower than that in the model group (*P* < 0.01, *P* < 0.05), but there was no significant difference in p47phox protein levels among the model, DCXD-5, DCXD-2.5, and AML groups.

## 4. Discussion

DCXD is a traditional Chinese herbal formula that has long been used in the clinic to treat chronic cardiovascular disease. The current study's major findings were that in DOCA-salt hypertensive rats, 4 weeks of DCXD treatment significantly reduced the arterial BP and heart-kidney target organ damage, inhibited abnormal sympathetic activity, improved brain sodium sensitivity and ENaC function, prevented the hypothalamus superoxide anion level and NADPH oxidase activity, and decreased the expression of NADPH oxidase subunits (p67phox and Rac1) and ENaC in the hypothalamus in a dose-dependent manner. These findings suggest that DCXD's BP-lowering and sympathoinhibitory effect on salt-sensitive hypertension may be due to the modulation of brain sodium sensitivity and ENaC function, as well as the inhibition of ENaC and NADPH oxidase expression in the hypothalamus.

Salt sensitivity is one of the most important intermediate manifestations of hypertension, which is defined by elevated arterial blood pressure. A common salt-sensitive model of hypertension characterized by neurohumoral activation is an animal model induced by chronic DOCA administration, reduced renal mass, and a high salt diet. Evidence indicated that DOCA-salt hypertensive rats were developed with an abrupt BP increase during the first 48 h followed by a slower BP rise over the next few weeks, resulting in persistent hypertension and target organ damage [17, 20]. Consistently, our study demonstrated that model rats exhibited severe hypertension and pathological damage of the heart and kidney observed in 7 weeks in comparison to that in the sham rats, while the DCXD treatments significantly improved those parameters. These results suggest that the DOCA-salt hypertensive rat model could be a reliable model to study the mechanism and intervention of hypertension target organ damage.

Sympathetic overactivity plays a crucial role in the development of salt-sensitive hypertension and related organ damage. Modulation of sympathetic overactivity is regarded as an important strategy for improving hypertension and its complications [3]. In the present study, the sympathetic activity was evaluated by indirect means with plasma catecholamines and BP responses to ganglionic blockade. The results indicated that DOCA-salt hypertensive rats exhibited elevated plasma NE levels compared with normotensive rats, and DCXD treatment suppressed the elevated plasma NE in DOCA-salt hypertensive rats. Furthermore, the ganglionic blocker hexamethonium was used to evaluate resting sympathetic vasomotor tone. Hexamethonium is a nicotinic cholinergic antagonist that acts in autonomic ganglia [21]. The acute depressor effect of hexamethonium was used to index the contribution of sympathetic activity in all groups. Our results showed that DOCA-salt rats had a greater fall in the BP level after ganglionic blockade than in the control group and that DCXD treatment attenuated those alterations. These results suggest that DOCA-salt-induced hypertension is dependent on a greater sympathetic activation, and the hypotensive effect of DCXD treatment is associated with inhibition of sympathetic nerve activity.

The PVN is a pivotal central site in the hypothalamus responsible for regulating sympathetic tone and extracellular fluid balance. Its parvocellular preautonomic neurons directly control sympathetic nerve activity, and the magnocellular neurons synthesize vasopressin and oxytocin in response to hyperosmolarity [22]. Numerous studies have demonstrated that enhanced neuronal activation of the PVN is an important mechanism for the acquisition of sodium sensitivity and abnormal sympathetic activity in the brain of salt-sensitive hypertensive rats [17, 19, 20]. To clarify the role of DCXD treatment on brain sodium sensitivity in DOCA-salt hypertensive rats, we investigated the effects of high-Na in the CSF on arterial BP and HR after ICV infusion of high-Na aCSF. It was shown that ICV injection of high-Na aCSF caused significantly greater increases in arterial BP and HR in DOCA-salt rats than in sham rats and that DCXD treatment prevented those alterations. This finding suggests that DCXD's sympathoinhibitory effect may be achieved by restraining the increase in sodium responsiveness within the brains of DOCA-salt hypertensive rats.

ENaC is expressed in areas of the brain related to fluid and electrolyte balance, sympathetic activity, and hypertension. Baseline ENaC expression within this region was higher in salt-sensitive hypertensive rats than in Sprague–Dawley rats and increased further with a high salt diet [12, 13]. Studies using ENaC inhibitors provide evidence of ENaC's role in the central response to a high salt diet and that the channel can perhaps sense small increases in the extracellular sodium and contribute to hypertension [13]. The present study used benzamil to assess the role of ENaC in the brain on cardiovascular responses following chronic DCXD treatment in salt-induced hypertension. The data demonstrated that ICV injection of benzamil significantly decreased MAP and HR in model rats compared to control rats, and the changes of MAP and HR in the DCXD-5 group were significantly lesser than that in the model group. Furthermore, the brain expression of ENaC protein in the model group was significantly higher than that in the sham group and that DCXD treatment attenuated those alterations. These findings imply that the DCXF treatment of salt-induced hypertension involves attenuating the expression and function of upregulated ENaC proteins in the brain.

The production of reactive oxygen species (ROS) is thought to be the key mechanism of ENaC activation in salt-sensitive hypertension [13, 23]. Numerous studies have found that increased brain ROS levels are necessary for increased sympathetic activity in response to high salt intake, and NADPH oxidase has been identified as the major source of ROS that is widely distributed in the brain [23]. NADPH oxidase is an enzyme complex that includes membrane subunits p22phox and gp91phox as well as cytoplasmic subunits p47phox, p67phox, and p40phox and the small G protein Rac1. Following hypertension, the expression and activity of NADPH subtypes in neurons and vessels increase dramatically, and inhibiting NADPH oxidase has a protective effect on the brain [15, 16]. Furthermore, NADPH oxidase-mediated ROS has been shown to act upstream of ENaC activation [16, 24]. The activity and subunit expression of NADPH oxidase in the hypothalamus were evaluated to investigate the potential role of NADPH oxidase in the inhibitory effects of DCXD treatment on brain ENaC activity. In our study, DCXD treatment significantly reduced the elevated NADPH activity and p67phox and Rac1 expression in the hypothalamus of DOCA-salt hypertensive rats. These findings suggest that DCXD treatments inhibit brain ENaC activity in part by reducing the increased ROS production mediated by p67phox and Rac1.

GAPDH and *β*-actin are the two most widely used housekeeping genes in Western blot. A number of studies have shown significant variation in some housekeeping genes both at the mRNA and protein levels in several disease states, such as alcoholic hepatitis, steatosis, and kidney tumor [25]. However, when evaluating the expression of specific proteins in brain tissues of hypertensive models by Western blot, the use of either GAPDH or *β*-actin for loading control has been reported in a considerable number of studies [26–28], suggesting that there is a consistency in the expression of GAPDH and *β*-actin in brain tissue, which is also consistent with the research results of Bauer et al. [29]. In the current study, due to the limitation of experimental conditions, *β*-actin was used for loading control to check the expression levels of p67phox and ENaC, while GAPDH was used for p47phox and Rac1 in the hypothalamus under independent experiments. The results showed no significant difference between GAPDH and *β*-actin among the experimental groups. In addition, we tried to keep all samples loaded with the same amount of total protein during the experiments. Therefore, the quantitative analysis of protein expression is more reliable in this study.

One source of concern is the potential chemical constituents and active ingredients in DCXD for the treatment of hypertension. It is a traditional Chinese herbal formula consisting of an aqueous extract of chuanxiong rhizoma and *Gastrodia elata*. Several phytochemical studies of DCXD showed that the main ingredients of DCXF were phenols, organic acids, phthalides, and nitrogen-containing compounds, which included gastrodin, ferulic acid, parishin, parishin B, parishin C, senkyunolide I, senkyunolide H, ligustilide, and 6,7-dihydroxyligustilide [6, 30]. Among those, ferulic acid was identified as the main active ingredient and marker component for quality control of chuanxiong rhizome in Chinese Pharmacopeia [31]. Accumulating evidence suggest that ferulic acid has a variety of biological activities, especially including antioxidant, anti-inflammatory, free radical scavenging, and cardiovascular protection effects [31]. In addition, gastrodin is known as the main bioactive constituent of Gastrodia elata, and possesses multiple neuroprotective effects including antioxidative, anti-inflammatory, regulating neurotransmitters, and inhibiting microglial activation. [32]. Several studies have also revealed that ferulic acid, gastrodin, and 6,7-dihydroxyligustilide were DCXD marker constituents for quality control or pharmacokinetic studies [33]. Thus, ferulic acid and gastrodin are likely to be the main ingredients of DCXD for lowering blood pressure and inhibiting sympathetic activity in hypertensive rats. Future research will focus on the qualitative analysis of chemical constituents and the identification of active ingredients of DCXD in hypertensive models.

Amlodipine (AML) is a dihydropyridine calcium-channel blocker widely used for the treatment of hypertension and ischemic heart disease in the clinic. In this study, we compared the effects of DCXD and AML on arterial BP, sympathetic activity, and brain sodium sensitivity in DOCA-salt hypertensive rats. AML is shown to cause comparable BP-lowering effects in DOCA-salt hypertensive rats. Interestingly, AML as a positive control has no significant ameliorative effect on abnormal sympathetic activity, brain sodium sensitivity, and ENaC function in DOCA-salt rats. This suggests that the antihypertensive effects of AML may be through vascular smooth muscle relaxation and arterial dilatation, and DCXD exhibits better advantages for improving brain salt sensitivity and abnormal sympathetic activity in treating salt-sensitive hypertension.

## 5. Conclusions

In summary, DCXD treatment reduced arterial BP and heart-kidney target organ damage, inhibited overactive sympathetic activity, improved brain sodium sensitivity and ENaC function, prevented hypothalamic superoxide anion level and NADPH oxidase activity, and decreased the expression of NADPH oxidase subunits (p67phox and Rac1) and ENaC in the hypothalamus of DOCA-salt hypertensive rats. These findings suggest that DCXD's BP-lowering and sympathoinhibitory effect on salt-sensitive hypertension may be due to the modulation of brain sodium sensitivity and ENaC function, as well as the inhibition of ENaC and NADPH oxidase expression in the hypothalamus. This study provides new laboratory evidence for further investigation of the mechanism of action of DCXF treatment on hypertension.

## Figures and Tables

**Table 1:** Effects of DCXD on heart index, kidney index, creatinine clearance, and urine protein in DOCA-salt hypertensive rats.

Parameter	Sham group	DOCA-salt group	DCXD-5 group	DCXD-2.5 group	AML group
HW/BW (mg/g)	3.11 ± 0.13	3.86 ± 0.11^*∗∗*^	3.42 ± 0.05^#^	3.63 ± 0.11	3.47 ± 0.06
KW/BW (mg/g)	5.1 ± 0.10	6.48 ± 0.18^*∗∗*^	5.53 ± 0.22^##^	5.66 ± 0.16^#^	5.73 ± 0.22^#^
Creatinine clearance (ml/min)	2.22 ± 0.26	0.47 ± 0.13^*∗∗*^	2.02 ± 0.31^##^	1.34 ± 0.33	1.07 ± 0.26^#^
Urine protein (mg/day)	4.21 ± 1.15	15.73 ± 3.44^*∗∗*^	7.62 ± 0.62^#^	10.31 ± 0.57	8.46 ± 0.41^#^

HW/BW, heart weight/body weight ratio; KW/BW, kidney weight/body weight ratio; AML, amlodipine. Data are presented as mean ± SEM (*n* = 6). ^*∗∗*^*P* < 0.01 vs. the sham group; ^#^*P* < 0.05 and ^##^*P* < 0.01 vs. the DOCA-salt group.

## Data Availability

The data used to support the findings of this study are available from the corresponding author upon request.

## Abbreviations

DCXD: Da-Chuan-Xiong Decoction
DOCA: Deoxycorticosterone acetate
AML: Amlodipine
BP: Blood pressure
BBB: Blood-brain barrier
ENaC: Epithelial sodium channel
NADPH: Nicotinamide adenine dinucleotide phosphate
PVN: Paraventricular nucleus
NE: Norepinephrine
UPLC-Q/TOF-MS: Ultra-performance liquid chromatography to quadrupole time-of-flight mass spectrometry
RSNA: Renal sympathetic nerve activity signals
ICV: Intracerebroventricular
SBP: Systolic blood pressure
DBP: Diastolic blood pressure
HR: Heart rate
MAP: Mean arterial pressure
ROS: Reactive oxygen species
aCSF: Artificial cerebrospinal fluid.
