# Supplementary material for: Da-Chuan-Xiong Decoction Ameliorates Sodium Sensitivity and Plasma Norepinephrine via Attenuation of Brain Oxidative Stress in the DOCA-Salt Hypertensive Rats
Source: Int J Hypertens. 2024 Feb 2;2024:2226143. doi: 10.1155/2024/2226143 (PMC10857883; doi:10.1155/2024/2226143)

Legend for Figure S1. Components analysis of DCXD by UPLC-Q/TOF-MS. (A) Total ion current chromatogram under negative ion mode. (B) Total ion current chromatogram under positive ion mode. (C) UPLC UV chromatogram of DCXD sample (UV 280 nm).

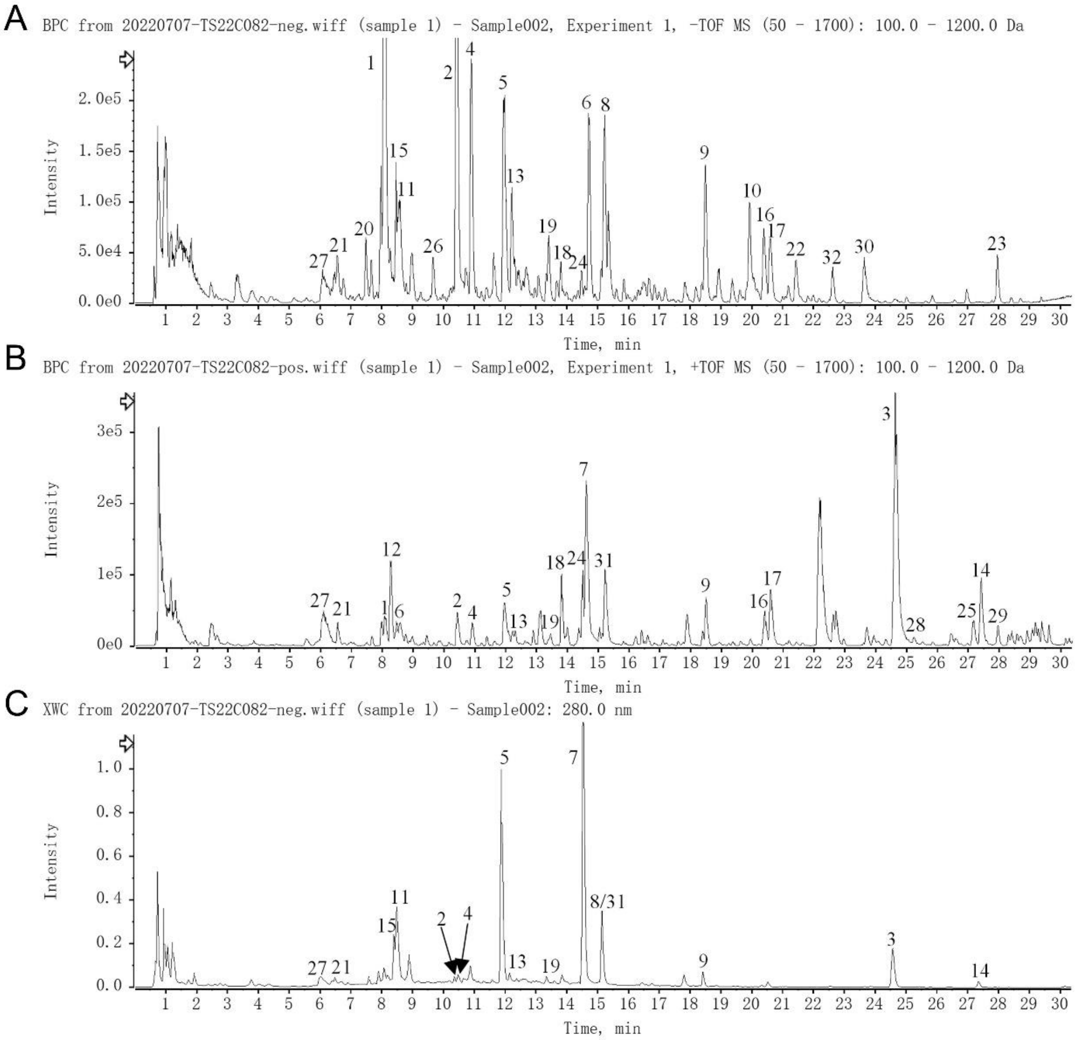

Supplement: Supplementary Materials — Additional file 1. Figure S1. Additional file 2. Table S1. [file 2226143.f1.zip › Figure S1.pdf]
