# Supplementary material for: Da-Chuan-Xiong Decoction Ameliorates Sodium Sensitivity and Plasma Norepinephrine via Attenuation of Brain Oxidative Stress in the DOCA-Salt Hypertensive Rats
Source: Int J Hypertens. 2024 Feb 2;2024:2226143. doi: 10.1155/2024/2226143 (PMC10857883; doi:10.1155/2024/2226143)
Supplement: Supplementary Materials — Additional file 1. Figure S1. Additional file 2. Table S1. [file 2226143.f1.zip › Table S1.pdf]

Table S1. Identification Results of Main Components of DCXD Samples

Num.	T(min)	adduct ions	*m/z* real value	*m/z* theoretical value	ppm	MF	mol wt	Name	DC	assignment
1	8.09	$[M-H]^-$	459.1175	459.1144	6.7	$C_{19}H_{24}O_{13}$	460.12	Parishin E	√	Tianma
2	10.43	$[M-H]^-$	727.2150	727.2091	8.1	$C_{32}H_{40}O_{19}$	728.22	Parishin B	√	Tianma
3	24.65	$[M+H]^+$	193.1232	193.1223	4.6	$C_{12}H_{16}O_2$	192.12	Senkyunolide A	√	Chuanxiong
4	10.91	$[M-H]^-$	727.2133	727.2091	5.8	$C_{32}H_{40}O_{19}$	728.22	Parishin C	√	Tianma
5	11.97	$[M-H]^-$	193.0517	193.0506	5.5	$C_{10}H_{10}O_4$	194.06	Ferulic acid	√	Chuanxiong
6	14.72	$[M+FA-H]^-$	549.2224	549.2189	6.4	$C_{23}H_{36}O_{12}$	504.22	Chuanxiongoside B		Chuanxiong
7	14.62	$[M+H]^+$	225.1127	225.1121	2.5	$C_{12}H_{16}O_4$	224.10	Senkyunolide I	√	Chuanxiong
8	15.22	$[M-H]^-$	237.0781	237.0768	5.3	$C_{12}H_{14}O_5$	238.08	Trimethoxycinnamic acid		Chuanxiong
9	18.50	$[M-H]^-$	577.1398	577.1352	8.1	$C_{30}H_{26}O_{12}$	578.14	4-O-8',5'-5''-dehydrotriferulic acid		
10	19.30	$[M-H]^-$	329.2346	329.2333	3.8	$C_{18}H_{34}O_5$	330.24	Trihydroxyoctadecenoic acid		Chuanxiong
11	8.58	$[M-H]^-$	167.0363	167.0350	7.8	$C_8H_8O_4$	168.15	Griffonilide	√	
12	8.28	$[M+H]^+$	414.1345	414.1329	3.7	$C_{17}H_{23}N_3O_7S$	413.13	S-(4-hydroxybenzyl) giutathione		Tianma
13	12.22	$[M+FA-H]^-$	1041.3167	1041.3093	7.1	$C_{45}H_{56}O_{25}$	996.31	Parishin A	√	Tianma
14	27.42	$[M+H]^+$	191.1081	191.1067	7.6	$C_{12}H_{14}O_2$	190.10	Ligustilide	√	Chuanxiong
15	8.47	$[M-H]^-$	353.0889	353.0878	3.1	$C_{16}H_{18}O_9$	354.10	Chlorogenic acid	√	Chuanxiong

16	20.41	[M-H]$^-$	205.0882	205.0870	5.8	$C_{12}H_{14}O_3$	206.09	(Z)-6,7-epoxyligustilide		Chuanxiong
17	20.60	[M-H]$^-$	207.1040	207.1027	6.4	$C_{12}H_{16}O_3$	208.11	Senkyunolide G		Chuanxiong
18	13.82	[M+H]$^+$	679.5127	679.5117	1.5	$C_{36}H_{66}N_6O_6$	678.50	Cyclohexa（iso）leucyl		Tianma /Chuanxiong
19	13.42	[M-H]$^-$	515.1232	515.1195	7.2	$C_{25}H_{24}O_{12}$	516.13	Isochlorogenic acid C	√	Chuanxiong
20	7.49	[M-H]$^-$	459.1180	459.1144	7.8	$C_{19}H_{24}O_{13}$	460.12	Parishin G		Tianma
21	6.56	[M-H]$^-$	515.1435	515.1406	9.0	$C_{22}H_{28}O_{14}$	516.15	Dicaffeoylquinic acid		Chuanxiong
22	21.44	[M-H]$^-$	205.0879	205.0870	4.3	$C_{12}H_{14}O_3$	206.09	(E)-6,7-epoxyligustilide		Chuanxiong
23	27.97	[M-H]$^-$	397.2039	397.2020	4.7	$C_{24}H_{30}O_5$	398.21	6,9-Ethanonaphtho[1,2-c]furan-8-carboxylic acid, 3-butylidene-1,3,4,5,5a,6,9,9a-octahydro-1-oxo-9-(1-oxopentyl)-, (3Z,5aS,6S,9S,9aS)-(ACI)		Chuanxiong
24	14.50	[M+H]$^+$	792.5953	792.5957	-0.5	$C_{42}H_{77}N_7O_7$	791.59	Cyclohepta（iso）leucyl		Tianma / Chuanxiong
25	27.17	[M+H]$^+$	195.1385	195.1380	2.8	$C_{12}H_{18}O_2$	194.13	Neocnidilide	√	Chuanxiong
26	9.67	[M-H]$^-$	401.1464	401.1453	2.7	$C_{18}H_{26}O_{10}$	402.15	Icariside F2		Chuanxiong
27	6.11	[M+H]$^+$	205.0976	205.0972	2.2	$C_{11}H_{12}N_2O_2$	204.09	Tryptophan	√	Tianma / Chuanxiong
28	25.25	[M+H]$^+$	191.1075	191.1067	4.4	$C_{12}H_{14}O_2$	190.10	3-Butylphthalide	√	Chuanxiong
29	27.96	[M+H]$^+$	191.1075	191.1067	4.4	$C_{12}H_{14}O_2$	190.10	Ligustilide		Chuanxiong

30	23.71	$[M-H]^-$	277.1454	277.1445	3.1	$C_{16}H_{22}O_4$	278.15	Senkyunolide M		Chuanxiong
31	15.22	$[M+Na]^+$	247.0953	247.0941	4.9	$C_{12}H_{16}O_4$	224.10	Senkyunolide H	√	Chuanxiong
32	22.63	$[M-H]^-$	277.1453	277.1445	2.8	$C_{16}H_{22}O_4$	278.15	Senkyunolide Q		Chuanxiong

Abbreviations: MF, molecular formula. mol wt, molecular weight. DC, database comparison.
